# Long-Term Experiences of Basic Education in Laboratory Animal Science

**DOI:** 10.3390/ani15111541

**Published:** 2025-05-25

**Authors:** Valeria Küller, Johannes Schenkel

**Affiliations:** German Cancer Research Center W30, Im Neuenheimer Feld 280, D-69120 Heidelberg, Germany

**Keywords:** basic LAS training, dummies, EU Directive 2010/63, EU Function A/C/D, evaluation, FELASA, GV-SOLAS, Miller’s pyramid, surplus animals, 3Rs

## Abstract

Adequate education in laboratory animal science and subsequently the attendance of relevant courses are mandatory prerequisites for animal experimentation. The course content for different stakeholders is stipulated by European and national regulations. If all of this content is covered, accreditation by competent bodies is possible and recommended. Here, we present our experiences with an EU-Function A/C/D accredited course (practical training with mice and rats) and an introductory seminar for undergraduate students, which have been running for more than ten years. All courses were organized in-house and were very relevant to the students and their needs but were also very labor intensive. The courses were systematically (and retrospectively) evaluated, showing a high degree of satisfaction and a great acquisition of knowledge, and the organizer was able to re-adjust the courses as needed over the years. Tests demonstrated the students’ progress and highlighted some parts of the lessons that were difficult to convey, such as those on legal regulations, housing and feeding, transport, GM animals, breeding, and the classification of severity. Dummies were proven to be very helpful at the beginning of the training but could not fully replace training with live animals. On-site lectures were favored over online sources, which were needed due to the pandemic. High standards in education are mandatory, and the accreditation process allows for the transferal of certificates to other institutions.

## Simple Summary

To adequately convey the fundamentals of laboratory animal science, a basic course accredited by competent bodies was offered in-house for more than ten years. Here, we present our experiences, the improvements made to the course following evaluations, the general preference for lectures presented on-site (not online), and our handling of special circumstances, such as the pandemic. Our data show that dummies might only be used successfully at the beginning of practical training. Analyses of the mandatory exam help to understand the extent of knowledge transfer and to identify which modules are obviously difficult to learn. Since laboratory animal science is not part of the curriculum, a corresponding seminar was offered to students of biology at the beginning of their studies, leading to a significant acquisition of knowledge. Follow-up surveys showed the sustainability of all the courses presented. The aim of this study is to share our experiences on how to offer, handle, and improve basic education in laboratory animal science with others, as well as to show the benefits of international accreditation, including the better transfer of knowledge and skills to a high standard.

## 1. Introduction

The appropriate education of all persons involved in animal experimentation is (not only legally, but also ethically) mandatory before starting their work. A legal framework was stipulated in European Union member states by the Directive on the Protection of Animals Used for Scientific Purposes 2010/63/EU [1] and, subsequently, national legislation. The curriculum consists of four functions, namely Function A (carrying out procedures on animals), B (designing procedures and projects), C (taking care of animals), and D (killing animals).

The content of these basic courses is given in [2] under the surveillance [3,4] of the European Commission via a working document and is described by the Federation of European Laboratory Science Associations (FELASA, www.felasa.eu [5,6,7]). Similar guidelines are published by national societies of laboratory animal science (LAS); e.g., the German Gesellschaft für Versuchstierkunde (GV-SOLAS, www.gv-solas.de) [8,9]. However, the EU member states are responsible for providing an appropriate education. Since many scientists and others move frequently, it is very helpful if their education on LAS and other subjects is accepted by their future employers and the Competent Authorities in charge (i.e., to ensure the portability and stackability of their education following the successful attendance of an accredited course). Therefore, the content of these courses is to be standardized, and the best way to check that a course complies with the requirements is by checking that it has been recognized by a national or (preferably) international body.

Before the course reported here was accredited by the FELASA (originally as an FELASA Category B course [7]), it had been acknowledged by GV-SOLAS, originally under less strict requirements. For the accreditation by the FELASA, we had to meet all of the FELASA’s defined requirements [5,7]. The evaluation of courses in general was/is an in-house policy; the course organizer as well as the faculty learned a lot from the comments of the attendees [10]. This resulted in several improvements.

The aim of a given course is sustainable knowledge transfer with assessable learning outcomes, such as those published, e.g., by the Education and Training Platform for Laboratory Animal Science (ETPLAS) [11,12]. Tests are a valuable tool to examine this but are also helpful to identify content that is difficult to convey.

LAS courses must meet the 3Rs as postulated by Russell and Burch (replace, reduce, and refine animal experiments) [13,14]. Strategies must be used that consider how to reduce the use of animals in the practical parts of education [15,16,17], but adequate training must also be guaranteed. An option is to replace at least part of the training on live animals with dummies. Several dummies are on the market that are suitable for different purposes. Evaluations of the dummies used in training courses help us to understand their practicability [18,19,20].

Students performing animal testing as part of their BSc or MSc thesis in life sciences or their doctoral thesis in human medicine must attend an LAS course. Many of them are unexperienced in LAS, resulting frequently in major problems within the course or the experimental part of their thesis [21,22]. However, LAS is not a mandatory part of these study courses. To overcome or mitigate these serious issues, an optional seminar was offered to students of biology in their second year, covering the theoretical modules of a Function A course [21].

The aim of this study is to share our experiences on how to offer, handle, and improve basic education in laboratory animal science with others as well as to show the benefits of international accreditation, including the transfer of knowledge and skills to a high standard.

## 2. Materials and Methods

### 2.1. Course Accreditation

The course was accredited and re-accredited on a national (GV-SOLAS [8,9]) and international (FELASA [5,6,7]) level. Both bodies have different accreditation schemes: GV-SOLAS asks for many details of the course, including all presentations, details on the practical program, the lecturers and trainers, as well as evaluations of previous courses. Applying for a (preliminary) accreditation by FELASA, all details of the course contents must be presented. FELASA will audit the course for a final accreditation on-site about a year later; a detailed report must be given every year. General course contents are stipulated by the EU [2] and in detail regarding the different modules by the accrediting bodies. However, the CVs of all lecturers and instructors are reviewed by both bodies. The Course Director (CD) has discussed with the lecturers the contents of their contributions; these were presented to GV-SOLAS within the accreditation process and to FELASA within the audits. The CD was responsible that all learning outcomes were covered. In addition, according to the German legal regulations, the Competent Authority in charge has to acknowledge the parts using live animals.

The pdf versions of all lectures were forwarded electronically to the students some days before the start of the course; the scriptum explaining the techniques to be taught in the practical parts were handed out as a hard-copy at the beginning of the practical part. All lecturers were experts in their scientific field; instructors of the practical parts were experienced in LAS and had special training for the techniques to be taught in the course.

### 2.2. Evaluation of the Courses

All data were captured and analyzed continuously during or directly after the individual course.

All evaluations were anonymous. The formats and the questions asked were given by the regulations of the German Cancer Research Center (DKFZ) or the University of Heidelberg. The best return rate of the filled forms was achieved if printed forms to evaluate the lectures of the day were handed out in the morning. The filled sheets were collected at the end of the day. The practical parts were evaluated by the same scheme, the questionnaires were collected directly after the completion of the corresponding part or day. Due to the circumstances (pandemic) in 2020, a Moodle tool was used to evaluate the lectures.

All graded information was analyzed according to the rules of the in-house Department for Further Education (MS EXCEL based). Answers of “free” questions as prose text were analyzed by the CD and if possible categorized.

Due to the in-house regulations, the scores were according to the German school grades and depending on the applicable rules best: (1), worst: (5), or (6). This was not feasible for some questions.

### 2.3. Moodle

In 2020 (pandemic), no on-site lectures were possible. As an alternative, a Moodle-based, FELASA-accredited online course by the University of Copenhagen was offered, covering the modules being part of the accreditation of our course. Since some parts of the course concern in-house needs which are not part of the accreditation (e.g., safety regulations) a further, separate Moodle tool, version 3.9.X LTS was used. The latter covered additional lectures, evaluations, and tests. All registered students had individual password-protected access.

### 2.4. Retrospective Surveys

The questions of the retrospective surveys were uploaded on a DKFZ web tool (DKFZ-HIFIS Lime Survey) https://onlinesurvey.dkfz.de assessed on 9 March 2020. All former students were contacted with the e-mail-address used during the course/seminar and were asked to fill the questionnaire on a voluntary basis within four weeks.

These anonymous surveys have passed the DKFZ ethics and privacy committees.

### 2.5. Animals and Animal Free-Technics

All animals were kept under standard housing conditions. The health of the animals was monitored according to the FELASA recommendations [23].

If available, only surplus animals without experimental purposes were used for training; at the end of the reported time, about 100% [24]. None of these in-part genetically modified lines developed a mutation-related phenotype or suffered from a mutation-based burden.

Animals were sacrificed according to the legal regulations and as agreed with the accreditors and the Competent Authorities; see “Permits”-section [1].

To train basic techniques, especially with unexperienced beginners, in some courses the sustainability of dummies was evaluated: Mimicky-Mouse MM001 (VetTech Solutions Ltd, Congleton, UK; www.vet-tech.co.uk, accessed on 19 January 2025), Trixie 45735 (TRIXIE Heimtierbedarf GmbH & Co. KG, Tarp, Germany; www.trixie.de, accessed on 19 January 2025), Ikea Gosig Mus 701.454.72, and Ikea Gosig Ratta 501.536.94 (IKEA Deutschland GmbH & Co. KG, Hofheim-Wallau, Germany; www.ikea.com, accessed on 19 January 2025). Mimicky is very sophisticated with many tools; Trixie is a “pet toy” in the size of a small mouse. Mus and Ratta are stuffed animals.

### 2.6. Statistics

If applicable, the collected data were evaluated for means, median, and standard deviation.

### 2.7. Permits

All animal experiments were acknowledged by the Competent Authorities Regierungspräsidium Karlsruhe (for DKFZ) or Darmstadt (for the Paul-Ehrlich-Institute, Langen [PEI]), Germany and under surveillance of the in-house animal welfare committees. As mentioned above, the course was accredited by GV-SOLAS and FELASA.

Lectures were offered at DKFZ, practical at DKFZ and PEI.

## 3. Results

This report demonstrates the experiences on LAS-education over more than ten years.

### 3.1. Educational Concept

For the basic education on LAS, an accredited FELASA Category B (later EU Function A/C/D) was offered [5,6,7]. The main experimental species were mice and rats; chicken, frogs, and gerbils were used for demonstrations.

The lectures covering the contents of the corresponding modules were presented by outstanding experts in their scientific field; subsequently, the lectures were offered twice/year: one edition in German, the other in English. Practical parts were performed following the lectures in small groups (in German, English, French, and Greek) depending on the students’ needs. Language skills were queried with the application [25,26].

The direct observations of the students’ procedural skills (DOPS) were reported individually with score-sheets by the trainers, close to that what was in the meantime published by ETPLAS [27]. Continuous training of the instructors and meetings of the faculty before and after the courses guaranteed a high consistency of the course-staff. The students had to show the instructor that they are individually able to apply the following techniques without the help of others [28,29]:

- Restraining with both, one and two hands;
- Sexing;
- Health check (in alphabetical order): body condition (from obese to emaciated), body orifices, cheeks, ears, eyes, hunching, fur, nose, orbital tightening, skin, vaginal plugs (if available), whiskers;
- Marking (ear punch, ear tags, tattoos);
- Applications (two subcutaneous techniques, intraperitoneal, intravenous, oral gavage);
- Sampling blood (fours techniques: *Vena caudalis*, *Vena saphena*, *Vena temporalis superficialis*, final heart puncture);
- Anesthesia (inhalation, injection; stages and signs of anesthesia);
- Cervical dislocation;
- Dissection.

It is clear that an attendee following a basic course will not be an expert, but the application of these skills/techniques are the minimum to pass. If not, additional training was offered.

The background of the attendees was academic (from BSc students to senior scientists coming from abroad and being enforced by the Competent Authority to attend a LAS-course since they could not prove appropriate training). Others were apprentices in education as animal caretakers or biology laboratory technicians in the educational departments of the institutes organizing this course [30].

Extensive evaluations with a high response rate were conducted. 1790 students have attended the course.

To introduce students of biology in an early study phase into LAS, a LAS-seminar was offered (without practical part).

### 3.2. Evaluations of the Course

Evaluations of all parts of the course accord with the in-house regulations of DKFZ. Each lecture and practical was assessed for the contents and speaker. Additional (open) comments were welcome. The framework was evaluated, too. These questions were asked:

#### 3.2.1. Lectures

- Were all relevant topics covered?
- Were your expectations fulfilled?
- Did you gain new ideas?
- Were examples relevant and of practical use?
- Was the speaker competent?
- Was the time used intensively?

#### 3.2.2. Practical Part

- Were the relevant topics covered?
- Were your expectations fulfilled?
- Did you gain new ideas?
- Were examples relevant and of practical use?
- Can you use parts of the content for your work?
- Was the trainer competent?
- Was the time used intensively?
- Did you have enough opportunities for active participation?

#### 3.2.3. Frameworks

- External frameworks (room, media)
- Organizational frameworks (registration, organization)

The students evaluated all individual lectures, all practical parts, and sometimes special questions; e.g., the use of dummies. We observed a general satisfaction and indications for improvements, too. The evaluations were also helpful to respond better to specific needs.

**Figure 1:**
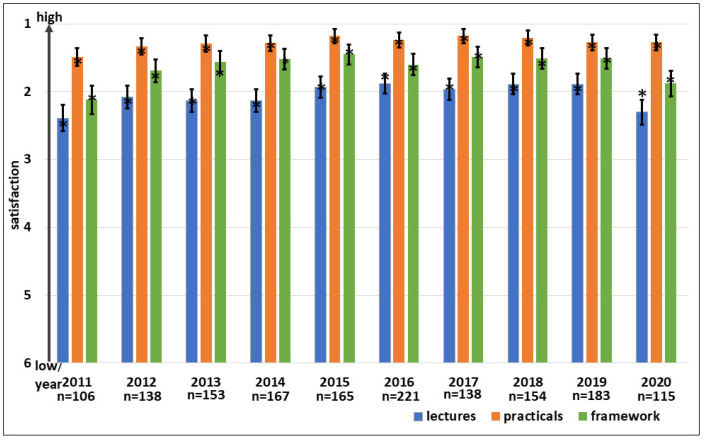
Summarized satisfaction with lectures, practical parts, and the general conditions; for details, see main text. The diagram for 2020 covers only the lectures of the in-house needs. The return rate was 80–100%, “1” is the best grade, “6” the worst. The bars indicate the standard error, the asterisks the medians.

A continuously increasing satisfaction was detected over the years except 2020 when many improvisations were necessary due to the pandemic measures; many decisions were only possible with a short notice, and subsequently the attendees (and the faculty) could not predict how things would turn out and had to be very flexible (Figure 1).

The practical parts in particular showed a high satisfaction; this might be due to the individual supervision by the instructors. In the case of lectures, the satisfaction was also high, but less than in the practical parts. This might be due to the large groups (up to 100 attendees) in a not-very-individual environment and some parts not so interesting for at least parts of the group of participants (see also further down and Table 1). It is noteworthy that the satisfaction was reduced in 2020 due to the COVID-19 measures. The frameworks showed after some starting troubles a high stability. Of course, in 2020, some participants were upset due to the circumstances.

In addition to the given questions, open comments were welcome. We tried to categorize these comments. The major outcome of these comments was the establishment of the English-speaking edition of the lectures and more languages in the practical parts. Other frequent comments were dealing with the contents of the course (without any chance of reducing parts due to the mandatory contents) or additional contents due to the increasing needs of information; e.g., the nomenclature of mutants. Many, often contradictory concerns were related to organizational issues.

### 3.3. COVID-19 Measures, Online Lectures

**Figure 2:**
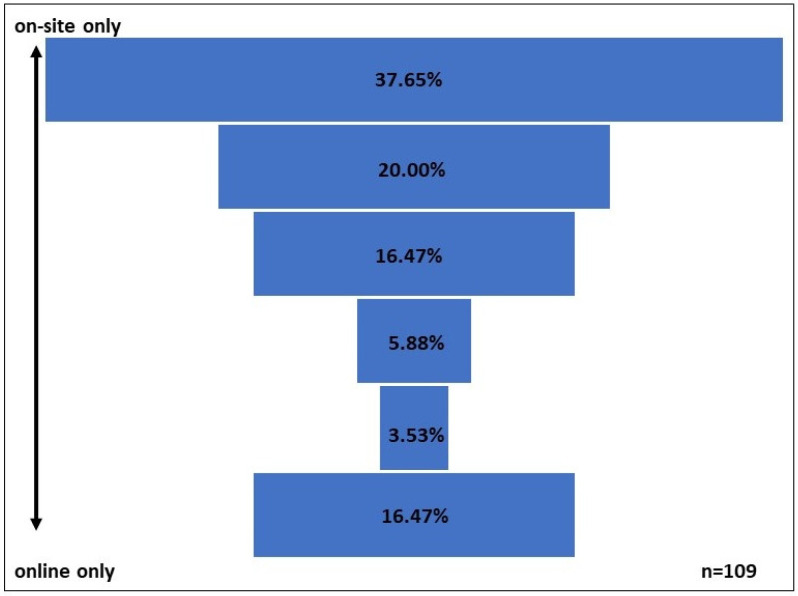
Online or on-site lectures: The majority of the 109 students attending the 2020 online editions of the course preferred a format of the course with lectures in an auditorium; online courses are less favored. The bars indicate from top to bottom the following preferences: 100% on-site; 80% on-site/20% online; 60% on-site/40% online; 40% on-site/60% online; 20% on-site/80% online; 100% online.

Due to the pandemic restrictions, all courses were stopped by March 2020. Since some students had to complete their thesis within a given time-span, we accepted, after an agreement with the accreditors and the Competent Authorities, the generous offer to attend the online lectures of a similar course presented by the University of Copenhagen. To address the local needs, some internal parts were added in a separate Moodle module (only the latter is part of the evaluations for 2020 in Figure 1). This platform was also used for the exam, as a discussion platform, and for the evaluations. Students were only accepted for the exam if all (anonymous) evaluations were uploaded. In this context, the students were asked about their preferences: online or on-site courses [31]. The majority (38%) of the 109 students attending these editions prefers on-site lectures (Figure 2), 16% prefer only online lectures, and 46% a mixture of both.

To make a head start, the practical parts were offered later in 2020 in a reduced format. To meet COVID-19 safety measures and to subsequently reduce the number of students and their density in the course laboratory, students had to state their immediate needs. In most cases, only the techniques of one species, often without surgery and euthanasia, were trained. A reduced certificate was issued confirming the successfully attended modules. In general, these editions of the course were not very satisfying, but were needed due to the circumstances.

### 3.4. Dummies to Replace Live Animals

**Figure 3:**
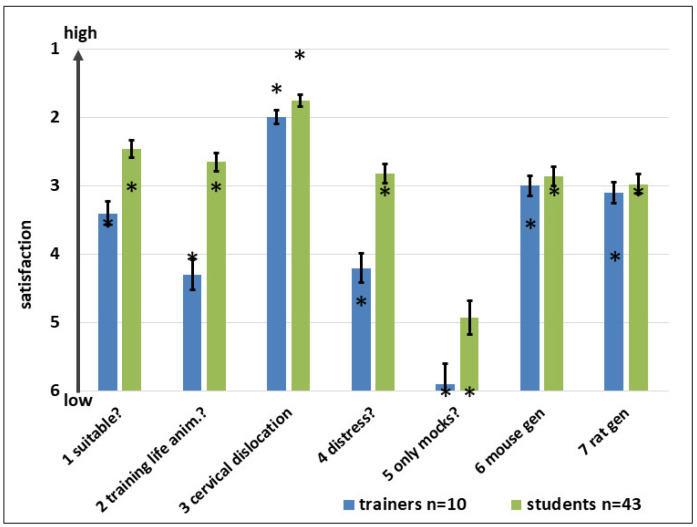
Dummies: The satisfaction of the use of dummies was given by trainers and attendees to introduce animal techniques for beginners but cannot replace the work with live animals completely. Ten instructors and 43 attendees were involved in this evaluation. “1” is the best grade, “6” the worst. The bars indicate the standard error; the asterisks the medians.

A longer-lasting discussion on animal welfare in education is the question of whether or to what extend animals might be replaced by dummies, especially for the training of beginners. To obtain a general impression of the suitability of dummies for training, several dummies were investigated, and the entire matter was evaluated (Figure 3). Taken together, students and trainers evaluated the use of dummies as very helpful at the beginning of the training, especially for very unexperienced attendees, but dummies cannot fully replace the use of live animals. Rather simple dummies were better rated than sophisticated items. Only a very few numbers of students preferred training only with dummies. In this context, the suitability of carcasses to train cervical dislocation was investigated and rated positively.

- 1 Are dummies suitable for training?
- 2 Is the use of dummies good training to handle live animals?
- 3 Is training on carcasses good training to perform cervical dislocation?
- 4 Does training with dummies reduce the distress for live animals?
- 5 Should only dummies be used in the practical part?
- 6 Summarized experiences with dummies (mouse)
- 7 Summarized experiences with dummies (rat)

### 3.5. Exam

The compulsory exam was scheduled between the lectures and the practical part. As agreed with the accreditors, each test had 32 multiple-choice questions (five answers, one correct) or easy-to-fill texts. At least 60% of correct answers were needed to pass. Altogether, 85% of the attendees passed with the first attempt; the others had a second attempt. Each test implemented 30% new questions.

However, to pass the practical parts, the active and successful participation of all parts was mandatory, and additional training was possible if needed. The direct observation of the students’ procedural skills (DOPS) was monitored [27]. To pass the practical part, the attendee had to be able to apply the techniques listed above. The pass was an individual decision by the instructor and, in difficult cases, by the CD, too.

The tests also help to understand learning progress. A pool of 400 questions was categorized to cover all modules. Analyzing the success rate of the question categories helped to identify difficulties for the students. We understood that the questions to some specific modules were more frequently incorrectly answered than others. A cut at 70% correctly answered questions was made and categorized to the corresponding modules. Following several internal checks showing that these parts were correctly taught, we identified some “problematic” modules (Table 1). Eye catching are legal regulations, housing and feeding, transport, details of GM animals, breeding, and the classification of severity.

**Figure 4:**
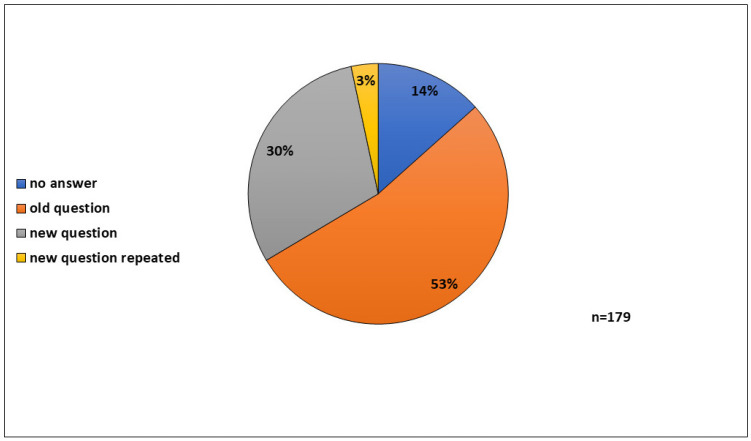
“Joker question”: 179 students had the chance to generate a new MC question; 54 students (30%) produced a new question (plus 3% multiply identical new questions); 95 students (53%) presented an already-existing question.

To offer the students an additional chance, to ask for the students’ creativity, to learn more about their state of knowledge, and to gain possibly additional questions, the option to generate a “joker question” was sometimes given. Therefore, students could develop a correct standard question (multiple-choice question: five answers, one correct); the bonus was counted like a passed question. In total, 179 students used the chance to generate a new MC question. A total of 54 students (30%) produced a new question (plus 3% multiply identical new questions). Not surprisingly, 95 students (53%) presented an already-existing question (Figure 4).

### 3.6. Retrospective Survey

**Figure 5:**
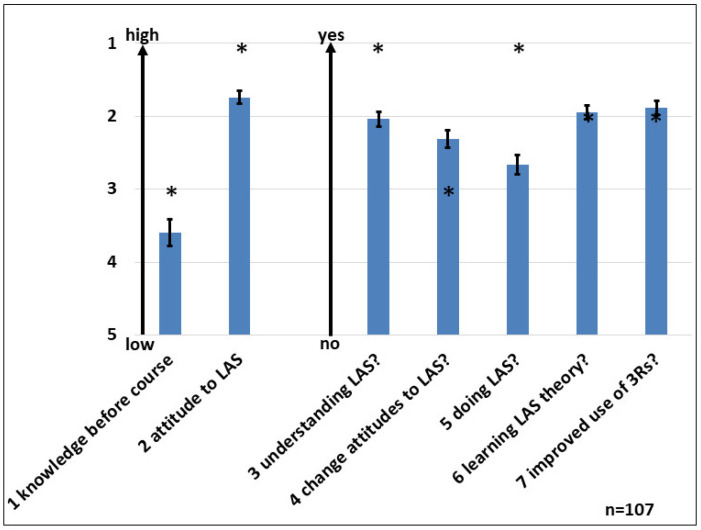
Retrospective course evaluation; 13.8% participation. Questions 1 and 2: “1” is the best grade, “5” the worst, questions 3 to 7: 1 is “yes”, 5 is “no”. The means are shown. The small bars indicate the standard error; the asterisks the medians.

To understand the long-term sustainability of the course, all 1790 former students were asked to fill an online questionnaire within a given time. Out of 786 deliverable e-mails, 107 former attendees responded, some of them many years after their course attendance, showing the sustainability of the course (Figure 5). Due to privacy regulations, when they attended the course is not known. The learning process within the course was shown. The responding former attendees stated that they had little knowledge on LAS at the beginning, but a good attitude before they participated. About 80% of them understood the aim and role of LAS better and acquired knowledge on it. However, about 70% positively changed their attitude towards LAS following the course (and possibly due to other, unknown reasons). The 3Rs (replacement, reduction, and refinement of animal experiments) and their use are a basis of LAS. It is therefore of major importance that the students were introduced to the idea of the 3Rs and that about 80% of the responders were obviously able to apply that [32]. Only 60% of them were meanwhile involved in animal experimentation.

- 1 What was your knowledge on LAS before the course?
- 2 What is your attitude towards animal experiments?
- 3 Does the course help to see LAS differentiated?
- 4 Was your attitude towards animal experiments changed by the course?
- 5 Are you involved in animal experiments after the course?
- 6 Was the course didactically helpful to obtain knowledge on LAS?
- 7 Was the course helpful to better apply the 3Rs in practice?

### 3.7. Seminar for Undergraduate Students of Biology

A seminar was offered for 15 years to 240 undergraduate students of biology within 15 terms. The aim of the seminar was to give some insights into LAS and to help the students to decide in which section of biology they wanted to become specialized [21]. To meet the study regulations, all students had to individually create a presentation on a specific topic (with support by the lecturer).

The contents were according to the lectures of a Function A course without the practical parts, but an animal facility visit and a video presentation of the techniques needed to generate mouse mutants were offered. It was evaluated with questions querying about the contents of the seminar, the learning progress, the understanding of LAS, the attitude towards LAS, and the framework with the opportunity of additional comments.

**Figure 6:**
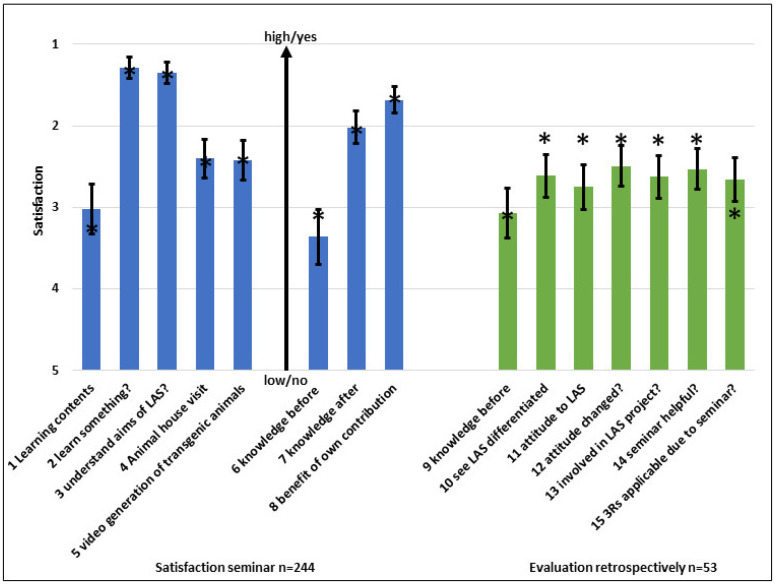
Biology students’ seminar: Evaluations (blue bars). Questions 1 to 5 indicate “1” as the best grade, “5” the worst. Retrospective evaluations (green bars). Questions 6 to 15: “1 is “high” or “yes”, “5” is “low” or “no”. The small bars indicate the standard error, the asterisks the medians.

However, at the beginning of the education on LAS, many students had no prospect as to what LAS means and often a negative view on it. It is important that the attitude towards LAS changed frequently with a better understanding of the role and aims of LAS. Of course, some students were not so happy with the content of the seminar, but specific parts such as the visit of the animal facility or the presentation of a video showing the major techniques needed to generate mouse mutants offered some new insights and were interesting for these students. The evaluations showed a high learning success in quite a new field for these students (Figure 6). The (mandatory) own presentation of an LAS topic was for the students time-consuming but obviously helpful for understanding. Over the years, no major changes in the evaluations of the students were observed (not demonstrated in detail).

All former students were asked to fill a retrospective questionnaire. Out of 76 deliverable e-mails, 41 former students responded, pointing to high long-term sustainability. Additionally, years after the seminar, the former students understood LAS in a more differentiated way. Some of them changed their attitude towards LAS. About 75% of the responders were in the meantime involved in animal experiments (Figure 6). Furthermore, about 75% reported that the seminar was helpful for the development of their studies and they learned how to apply the 3Rs, quite a new topic for them.

- 1 Are the learning contents interesting?
- 2 Did you learn something?
- 3 Did you understand the aims of LAS?
- 4 Was the animal house visit helpful to obtain more insights into LAS?
- 5 Was the video presentation on how to generate mutant animals helpful to obtain more insights into LAS?
- 6 What was your knowledge on LAS like before the seminar?
- 7 What was your knowledge on LAS like after the seminar?
- 8 Was the preparation of your own contribution to the seminar beneficial to understand (parts of) LAS better?

Retrospective evaluation seminar. Participation rate 69.7% (green bars)

- 9 What was your knowledge like on LAS before the seminar?
- 10 Did the seminar give insights to see LAS in a differentiated way?
- 11 What is your attitude towards animal experimentation?
- 12 Did the seminar change your attitude towards LAS?
- 13 Are you already involved in an LAS project?
- 14 Was the seminar helpful to better understand LAS?
- 15 Did the seminar make the 3Rs more applicable for you?

## 4. Discussion

An appropriate and sustainable education is a prerequisite for animal testing. Here, we report our experiences on the basic LAS education over more than ten years.

### 4.1. Course Settings, Concept, and Participants

The strict framework for those courses is stipulated by European and national regulations. The challenge for the course faculty was to handle with limited resources up to 200 attendees/year with at least two languages and to offer an interesting, but mandatory course under the given restrictions to quite a heterogenous audience, from apprentices in education to animal care takers, or biology laboratory technicians to experienced senior scientists. A high standard of all contributions, lectures as well as practical training, is very important. For the lectures, we also selected colleagues with outstanding experience in their scientific field from abroad. This was limiting the frequency to two editions/year. For the practical training, experienced instructors were selected; most of them were employed by DKFZ or PEI. They were familiar with the in-house settings and in many cases also with the needs of the students’ research groups, subsequently being able to support in case of specific questions or needs.

We also understood the importance that the CD was continuously available and supervising the course. It was easy to re-adjust if needed. This strategy resulted in several improvements. For the practical parts, it also keeps the opportunity to commit individual needs better, as well as under in-house conditions. However, this strategy is straight forward but also resource consuming and needs the cooperativeness of all stakeholders: lecturers, trainers, and the people supporting the course in the background.

To maintain high standards, lecturers and instructors must be motivated. Many of them did this as a part of their professional duties; external persons received a small salary and travel expenses according to the regulations of the public services. As a small appreciation, the university acknowledged these teachings as “academic teaching hours”, slightly helping to fulfil their academic teaching commitments.

**Figure 7:**
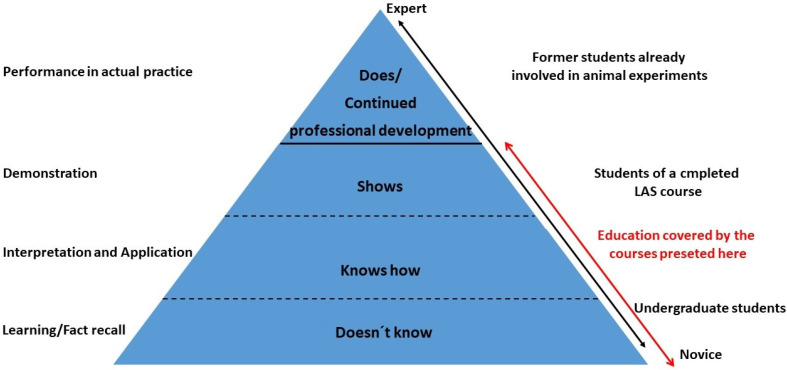
Development of the attendees in an adapted Miller’s Pyramid [33]. The red colored arrow and comments demonstrate the parts of education covered by a basic education; the performance in actual practice and CPD are not part of basic courses.

With this strategy, we were able to support the attendees to obtain knowledge on LAS to a certain level (Figure 7). However, when completing a basic course, nobody is experienced. This can only be achieved when continuously practicing these techniques, as well as in the framework of CPD. The other way around, it makes no sense to attend a course on LAS without working on animals afterwards [33].

The retrospective survey (Figure 5) showed that most of the participants majorly benefitted from the course. No deeply differentiated analyses were possible, but we learned repeatedly that former students changed their position (as well as to other countries). As far as we know, in all cases, their certificate was accepted by the new employer and the Competent Authorities. Most of these students were academic, but there were also a few technicians (former apprentices). However, this argues for the portability of certificates when attending an accredited course.

In the long-term, we understand that attendance of LAS-courses by apprentices is very helpful for their professional future. Within Europe, different training strategies have been established [1,2,5,22]. The successful completion of an accredited LAS course certifies a high educational standard and is, as we have learned from former apprentices, very helpful to be in a better position, including abroad.

Scientists, and more and more technicians too, have to be very flexible; i.e., to frequently change their position and subsequently the location, possibly connected with different legal regulations. If international standards are met and the candidate can show a certified education, these movements are easier. Therefore, it is of major importance that (not only) LAS courses are accredited, meeting some standards. Over the years, we have dealt with many colleagues from abroad; it was very easy to integrate them in the current (legal) framework if they had already attended an accredited course elsewhere. The other way around, we have had major issues with colleagues experienced with many years coming from other places and being unable to prove their attendance on a basic LAS course. They were forced by the Competent Authorities to participate on the basic course. Subsequently, there was little benefit since they were already familiar with most of the course contents and blocked spaces needed by others, which is not a satisfying matter. The strict legal regulations and some differences between European and German regulations were also a reason why we applied for both, the FELASA and the GV-SOLAS accreditation.

### 4.2. Exams

The exam does not only serve as learning control, it is also a tool to understand the difficulties of the students. After cross-checks with the lecturers and their presentations to exclude possible problems when transmitting knowledge, we identified some modules with an increased fail rate (Table 1). Courses must cover many topics as required by legal regulations, obligations of the accreditors, etc. However, not all students are interested in all contents. At least as shown by the test results, contents not very close to the students’ interests are sometimes hard to convey. Cross-checks with the satisfaction of the corresponding modules showed that there is a link between rather poorly rated modules and an increased fail rate.

The aim of a “joker question” was to induce the creativity of the students but also a tool to understand the circulation of already-asked questions, even if all tests were collected and not returned. However, despite all countermeasures, the circulation of test questions could not be omitted (Figure 4). Last but not least, this was also a tool to gain additional questions.

The development of practical skills (DOPS) was checked according to the list in Chapter 3.1. All students had sufficient time to train and, at the end, had to show to the instructor that they can perform these techniques at least at a beginner’s level. In case of difficulties, additional training was possible. It should be pointed out that beginners at the end of a basic course are introduced to these techniques. To become an expert, much more training, more experience, and CPD is required (Figure 7).

### 4.3. Dummies for Practical Training

A long-lasting matter is the use of live animals for teaching purposes and approaches to reduce at least the number of animals used for training [16,17,18,19,20]. We investigated some dummies at different parts of the course. Two of these dummies were sold by a furniture store with the advantage to wash, disinfect, and re-use it in later courses and the disadvantages of a relatively large size and limited availability. Another is a “pet toy” from zoo-shops with the advantage of a correct size of a mouse and a high availability. Here, the disadvantage is the limited option of cleaning and disinfection. These dummies cost about 2 EUR/item.

Students and trainers were happy with the inexpensive dummies for the initial training of basic techniques such as restraining, ear-punching, and cervical dislocation (in a second step with carcasses), even if the size of the dummies was close to a live mouse, especially for unexperienced students. However, in group discussions, it also became clear that this is only helpful at the very beginning or the introduction of new techniques. It will reduce the stress for the animal and student, but dummies cannot replace the work with live animals [34]. Best rated was “Trixie” (Figure 3). Alternatively, a very sophisticated and very expensive (1200 EUR/item) model with many more options was examined. This was not satisfying for our settings; it is too complex, and its options were used only to a small extent.

Importantly, depending on availability, only surplus animals were used as live animals for training purposes, due to optimized course planning up to 100% [24]. Both, dummies and surplus animals contribute to the 3Rs in training.

### 4.4. Evaluations

The feedback of the course was standardized with continuous evaluation helping to discover the attitude towards the course, and the usefulness and sustainability of the learning matters. Subsequently, we could also respond to the wishes of the students, at least in future courses. The described strategy to evaluate led to a high response rate and showed a high satisfaction with the course, increasing over the years. Rather formal topics such as legal and in-house regulations or safety aspects were rated continuously worse than topics closer to animal experiments (see summarizing Figure 1). An earlier report from Switzerland demonstrated similar satisfaction of the attendees [10].

A major consequence of the evaluations was the establishment of the English-speaking edition, in this case resulting from the option to add further comments. Originally, an English scriptum or English slides in German-speaking lectures were offered. This was not very satisfying, even since more and more international, non-German-speaking students attended the course. On the other hand, it was clear that some attendees can only be successfully educated in their native language of German. Thus, editions of the lectures in both languages were developed, even if only a few non-German-speaking students were expected. However, as the requirements changed, more and more English-speaking places were needed. The language issues were not so relevant in the practical parts, since the small group size allowed an ad hoc decision of the language. Practical courses were also offered in French and Greek. In addition, issues that were mentioned frequently could be adjusted, as more relaxed schedules, especially the time between lectures and exams, resulted from the evaluations. The availability of the course material [time and form (printed/electronic)] was also frequently addressed. Often, other organizational requests could not be fulfilled due to several restrictions; e.g., space, availabilities etc.

Some contents, mostly of the lectures, were repeatedly criticized. They were obviously boring for some attendees, but mandatory for the course; e.g., legal regulations, especially in the English-speaking courses with a relative high number of attendees not familiar with the European and/or the German legal systems. Other criticisms disappeared over the years since these parts were taught from the beginning, but later became more important for the individual work; e.g., the nomenclature of mutants, breeding strategies, types of mutants, feeding, or animal hygiene strategies.

Evaluating the additional comments showed that the students wanted to obtain more information in animal biology and all techniques to handle animals. Some topics should be discussed more concisely, such as databases, ethics, or in-house regulations.

The evaluation results were discussed within the faculty; updated lectures also led to improved acceptance.

The practical parts had, with a few exceptions, continuously very good ratings. Outstanding experienced trainers taught small groups of four or five students. Of course, some students did not pass the practical parts or discontinued their attendance. They were not so enthusiastic. Organizational issues were frequently cited reasons for discussions. A major point was the date of the practical part; a general issue due to the availability of course labs and instructors. If we understood a technical problem, we tried to solve it immediately. Fortunately, many students expressed thanks to their individual instructors in the evaluation sheet, since most trainers were also familiar with the needs of the students’ working groups. However, the faculty became over the years more and more experienced with the course and learned how to handle any issues.

It was challenging to contact former students for the retrospective survey; 44% were found. Out of these, 13.8% responded on a voluntary basis. At least for those interested in LAS, sustainable acquisition of knowledge on LAS, changes in the attitude towards animal experimentation, and an improved use of the crucial 3Rs were demonstrated [34,35]. Due to the obligations about the survey, we were not able to understand to which group of attendees (apprentices, senior scientists, etc.) the individual answer belonged. So far, some former students (40% of the responders to the retrospective survey) are not involved in animal experimentation (Figure 5). To avoid unnecessary participation, the prerequisites to attend an LAS course should be revised.

### 4.5. Pandemic Measures

Due to the pandemic restrictions, we tried to agree a stand-alone minimal course with the accreditors and the Competent Authorities. These reduced programs are not satisfying but a short-term solution under the given circumstances. However, they keep the danger of permanency [36,37]. This development is, from our understanding, undesirable. In addition, in basic education, more than the minimum needs should be taught. The students perform animal experiments after the course, and they therefore must develop an enormous responsibility; only high training standards meet these requirements. Intensive individual training is mandatory. This training needs individual mentoring and practical training. To our own surprise, most of the students attending the online course prefer on-site lectures, offering more possibilities to discuss topics of the course (Figure 2) [31]. It should be pointed out, that the circumstances in 2020 were extraordinary. The reduced course contents obeyed the current needs and should never become a standard.

### 4.6. Introductory Seminar for Undergraduate Students of Biology

The aim of this seminar was to introduce undergraduate students to LAS and to help them to make decisions for their scientific or professional future. The other way around, the attendance of the seminar should help to overcome the frequent issues that students understand not before performing their BSc or MSc thesis or under a time pressure whereby they do not want to or cannot perform animal experiments. It is important for sustainability that former students claim to be successfully introduced by this seminar to the basics of LAS. They can better understand and in a more differentiated way what LAS means. Some of them (75% of the responders to the retrospective survey) were in the meantime involved in animal testing (Figure 6).

The retrospective survey showed that the students who answered on a voluntary level had input from their studies by the seminar and were sensitized to all issues dealing with LAS.

## 5. Conclusions

To summarize, intensive education is a prerequisite for animal experimentation and animal welfare. To become familiar with these topics, education should start as early as possible. A sustainable education needs continuous feedback and re-adjustments, also according to the students’ needs. It must meet high standards, too. However, this basic education is an introduction and sensitization to the topic of LAS. It is the beginning of training. Nobody who has already completed the course is experienced on LAS or an expert. This must be developed by practical experience, training the techniques applied, and CPD (Figure 7).

According to our data, training with dummies or online lectures are helpful for some parts but cannot replace the work with live animals and on-site courses completely [18,19,20]. With respect of the major responsibility of all people involved in animal experimentation, extensive education/training is mandatory. Reduced courses as needed in the context of the pandemic should be omitted. Courses should be accredited by a competent body leading to stackability of the course and the portability of the certificate to other places as well as to avoid that experienced people attending a basic course. Course management of the course must be stable in the long term.

The training of students at an early state of their study and an intensive introduction into LAS before starting the practical work is a successful and sustainable strategy to sensitize the students and to improve animal experimentation. It might be very helpful if, at least in some basic study courses, an introductory seminar on LAS is available or, better, compulsory.

## Figures and Tables

**Table 1:** Questions to modules (according to the EU regulations) that were not answered correctly by at least 30% of the test attendees.

EU-Module	Title of Module	Sub-Module No.	Contents of the Sub-Module
EU-Module 1	National Core	1.1	National and EU law
		1.6, 1.7, 1.8	Local animal welfare bodies and national committee for the protection of animals
EU-Module 2	Ethics, Animal Welfare, and the 3Rs Core	2.1	Different views relating to the scientific uses of animals
		2.9	Severity classification system
EU-Module 3	Basic and appropriate biology. Species-specific (theory) Core	3.1.5	Dietary requirements
		3.1.6	Importance of enriched environment
		3.1.8	Alterations to the genome
EU-Module 4	Animal care, health, and management Core	4.2	Housing conditions
		4.5	Organisation of animal facilities
		4.6	Dietary requirements
		4.8	Methods for marking
		4.9	Risks in the animal facility
		4.10	Breeding programmes
		4.11	GM animals
EU-Module 5	Recognition of pain, suffering, and distress. Species-specific Core	5.5	Severity classification
EU-Module 6	Humane methods of killing (skills)	6.2.2	Confirmation of death
EU-Module 20	Anaesthesia for minor procedures	20.12	Post anaesthetic recovery
EU-Module 23	Devise appropriate breeding programmes for laboratory animals given specified conditions	23.17	Breeding programmes
	Explain the use and problems associated with genetically altered animals	23.22	GM animals in research
		23.24	Generation of GM animals
	Know procedures for the safe and legal transportation of animals	23.25, 23.26, 23.27	Safe and legal transportation of animals

## Data Availability

All data are available from the corresponding author.

## Abbreviations

The following abbreviations are used in this manuscript:

BSc	Bachelor of Science
CPD	Continued Professional Development
CD	Course director
DKFZ	German Cancer Research Center, Heidelberg, Germany
DOPS	Direct Observation of Procedural Skills
ETPLAS	Education and Training Platform for Laboratory Animal Science
EU	European Union
FELASA	Federation of European Laboratory Animal Science Association
GV-SOLAS	Gesellschaft für Versuchstierkunde/Society for Laboratory Animal Science
LAS	Laboratory Animal Science
MSc	Master of Science
PEI	Paul-Ehrlich-Institute, Langen, Germany
3Rs	Replacement, Reduction, Refinement
